# Effects of Acupuncture in Ischemic Stroke Rehabilitation: A Randomized Controlled Trial

**DOI:** 10.3389/fneur.2022.897078

**Published:** 2022-06-23

**Authors:** Lixia Li, Weifeng Zhu, Guohua Lin, Chuyun Chen, Donghui Tang, Shiyu Lin, Xiaorong Weng, Liqin Xie, Lihong Lu, Weilin Li

**Affiliations:** ^1^Department of Acupuncture and Moxibustion, The Affiliated TCM Hospital of Guangzhou Medical University, Guangzhou, China; ^2^Department of Acupuncture and Moxibustion, The First Affiliated Hospital of Guangzhou University of Chinese Medicine, Guangzhou, China; ^3^Department of Psychiatry, Liwan District Hospital of Chinese Medicine, Guangzhou, China; ^4^Xin Hua College of Sun Yat-sen University, Guangzhou, China

**Keywords:** acupuncture, ischemic stroke, randomized controlled trial (RCT), rehabilitation, clinical trial

## Abstract

**Background:**

Acupuncture is a well-known treatment option for ischemic stroke recovery, but evidence of its effectiveness remains limited. This is a randomized controlled trial to evaluate the effectiveness of acupuncture treatment for ischemic stroke rehabilitation.

**Methods:**

Rehabilitation training was provided to the control group. In acupuncture arm 1, these acupoints were derived from the ancient books, including GV20 (*baihui*), GV26 (*shuigou*), PC9 (*zhongchong*), ST6 (*jiache*), ST4 (*dicang*), LI15 (*jianyu*), LI11 (*quchi*), LI4 (*hegu*), GB30 (*huantiao*), GB31 (*fengshi*), GB34 (*yanglingquan*), and GB39 (*xuanzhong*). In acupuncture arm 2, the acupoints used were GV20 (*baihui*), PC6 (*neiguan*), LI11 (*quchi*), LI10 (*shousanli*), SJ5 (*waiguan*), LI4 (*hegu*), GB30 (*huantiao*), ST36 (*zusanli*), GB34 (*yanglingquan*), SP6 (*sanyinjiao*), ST41 (*jiexi*), and LR3 (*taichong*), which were extracted from *Acupuncture and Moxibustion Science*. After acupuncture, the needles were left in for 30 min and manually manipulated every 10 min. The three groups received treatment once a day, 5 times a week for 2 weeks. The primary outcome was the National Institutes of Health Stroke Scale (NIHSS), and the secondary outcomes were the Barthel Index (BI) and the Modified Ashworth Scale (MAS). Outcomes were measured in patients both before and after treatment.

**Results:**

A total of 497 patients with ischemic stroke were randomized into either arm 1 (159 cases), arm 2 (173 cases), or the control group (165 cases). After 2 weeks of treatment, the NIHSS scores for arm 1 were lower than those of the control group (*P* = 0.017); the BI scores were higher in arm two than that in the control group at T2 (*P* = 0.016) and follow-up (*P* = 0.020). Additionally, there was no significant difference between arm one and the control group for either the BI scores or the MAS scores (*P* > 0.05) and no significant difference between arm two and the control group for the MAS scores or the NIHSS scores (*P* > 0.05).

**Conclusion:**

The clinical efficacy of arm 1 and arm 2 (acupuncture groups) was superior to that of the control group, but there was no difference between the effects of the two acupuncture groups.

**Clinical Trial Registration:**

http://www.chictr.org.cn/index.aspx, identifier: ChiCTR-IOR-16008627.

## Background

Stroke is widespread around the world. According to recent reports (1–3) the incidence of stroke in China is 274–379 per 100,000 people, of which ischemic stroke accounts for 60–70%. Three-fourths of stroke survivors are left with disability of varying degrees and about 40% are severely disabled. In China, the care for patients with stroke imposes heavy economic burdens on both the state and many families.

Ischemic stroke is the common term for cerebral infarction, which disrupts cerebral artery blood flow through several pathways, such as cerebral arteriosclerosis or cerebral artery thrombosis, causing hypoxia and ischemic necrosis in local brain tissue. This results in corresponding neurologic deficits (4). The clinical manifestations of ischemic stroke are focal neurological deficits such as hemiplegia, aphasia, dysphagia, visual impairment, and mental disturbances. Ultra-early thrombolysis has been used widely in the acute phase of stroke. However, due to its time-restricted application, the probability of thrombolysis is only 2.4% (5). This leaves intravenous or oral medications, rehabilitation training, and prevention of complications as the primary treatment measures for most patients with stroke.

Acupuncture has been used for the treatment of stroke since ancient times. The stroke symptoms and acupoints were recorded in the earliest Chinese medicine canon named the Yellow Emperor's Inner Classics (Huang-Di-Nei-Jing in Chinese), while the syndrome differentiation of stroke and its treatment were described in the Comprehensive Achievements of Acupuncture and Moxibustion (Zhen-Jiu-Da-Cheng in Chinese). This provides a theoretical basis for the treatment of apoplexy by acupuncture. At present, new acupuncture theories and techniques are still being developed. These include body acupuncture, scalp acupuncture, electrical acupuncture, and eye acupuncture. Effective treatment is dependent on the proper choice of acupuncture points. The acupoints in this study were selected based on ancient literature and Acupuncture and Moxibustion Science (6). They were chosen because of their wide application, frequent use, and direct curative effect. These acupoints possess the following characteristics: they are connected to the yang meridians. The Du Meridian, the Large Intestine Meridian, the Stomach Meridian, and the Gallbladder Meridian are the most relevant yang meridians in this regard. Since GV20 is the meeting of various yang meridians, it is the most frequently selected acupoint, which has the effect of awakening the brain and pacifying the spirit (Xingnaoanshen) and expelling wind to open the orifices (Qufengkaiqiao) (7). The Stomach Meridian and the Large Intestine Meridian have the functions of harmonizing Qi and blood and relieving limbs and joints. The Gallbladder Meridian is related to tendons and has the function of stimulating the circulation of the blood and relaxing the muscles and joints. In Chinese medicine, yin and yang balance is important to maintain the physiological equilibrium of the human body. The acupoints on the yang meridians have the effect of replenishing yang qi. However, patient with apoplexy often shows symptoms such as paralysis and aphasia that belong to Yin syndrome. This is in line with the viewpoint of treating yin disease with Yang. The other feature of the selected acupoints in this study is that they have a unique therapeutic effect and a specific name. The main ones are the convergence points (Jiao-hui-xue) and the He acupoints, in which Jiao-hui acupoints enable the Qi and blood of meridians to communicate with each other for the treatment of the disease of intersecting meridians. The He acupoint is one of the five Shu points (Wu-shu-xue) and it is the acupoint with the greatest Qi and blood. Therefore, it is easier to mobilize Qi and blood in the treatment of apoplexy by needling such acupoints. This provides a sound theoretical basis for effective stroke treatment (8–10). However, acupuncture's clinical efficacy needs further validation in clinical trials.

A prospective randomized controlled trial was conducted to explore an effective scheme for acupuncture treatment of ischemic stroke and to provide scientific evidence for the effectiveness of acupuncture for stroke rehabilitation.

## Methods

### Settings and Subjects

This study was a randomized controlled trial, conducted by the Affiliated TCM Hospital of Guangzhou Medical University, the First Affiliated Hospital of Guangzhou University of Chinese Medicine, and the Liwan District Hospital of Chinese Medicine. In this study, patients were randomly divided into three groups: the ancient books acupoint group (treatment group 1), the modern literature acupoint group (treatment group 2), and the rehabilitation group (control group). The random assignment operation was completed by the personnel of the Key Research Laboratory of Clinical Research Methodology of Guangdong Hospital of traditional Chinese medicine using SAS version 9.2 software. At the same time, the personnel responsible for the efficacy evaluation will be hired separately and will not know the grouping of patients. The personnel for the final data analysis will also be hired separately and will not participate in the specific clinical implementation work and design scheme of the subject. Doctors and patients are aware of the study interventions. The study reporting was in compliance with the requirements of the CONSORT 2010 statement (11).

Patients were randomly divided into arm 1, arm 2, or the control group. The randomization was performed by personnel from the Key Research Laboratory of Clinical Research Methodology at the Guangdong Provincial Hospital of Chinese Medicine. SAS version 9.2 was used to complete the randomization. The trials were single-blind as the outcome evaluators and data analysts were unaware of the groupings. Neither patients nor the acupuncturists performing the interventions were blinded. To avoid bias, individuals responsible for the evaluation of the efficacy were hired separately and were unaware of patient groupings. Analysts did not participate in the clinical procedure or the design of the project. The control group used the basic treatment plan, including baseline medications and rehabilitation training.

The trial was conducted between July 2016 and July 2017 and patients who met the following criteria were included in this study:

(1) Diagnosed with the ischemic cerebrovascular disease by either CT or MRI (12);
(2) With a period of 2 weeks to 12 months after acute stroke;
(3) With a number of strokes ≤3;
(4) Aged between 40 and 75 years (men or women); and
(5) Had clear cognitive faculties, stable vital signs, no obvious dementia, no obvious hearing impairment, and ability to cooperate with rehabilitation training.

Exclusion criteria were those patients who:

(1) Had already received other treatments that were not part of this study plan;
(2) Suffered from transient ischemic attack or reversible ischemic neurological deficit (RIND);
(3) Had neurological deficit, which was not related to ischemic stroke;
(4) Suffered from mental disorders or other severe diseases;
(5) Had severe aphasia, sleep apnea, deafness, or severe cognitive impairment;
(6) Patients with severe heart, liver, kidney, and other important organ diseases;
(7) Those with diabetes or endocrine diseases. Complicated with serious lesions of important organs;
(8) Before onset, there are malnutrition diseases, intestinal diseases, or stress ulcers with bleeding; and
(9) Those who are unwilling to participate in this study or withdraw from this study.

### Treatment Process

The standards of this study met the requirements of the *Standards for Reporting Interventions in Clinical Trials of Acupuncture* (STRICTA) 2010 (13). Ethics approvals were obtained from the Ethics Committee of the Guangzhou Chinese Medicine Hospital, the First Affiliated Hospital of Guangzhou University of Chinese Medicine, and the Liwan District Chinese Medicine Hospital (Reference no. 2016NK001) before conducting this study. All the patients also received rehabilitation training (12–14). Rehabilitation training followed the guidelines of the *Rehabilitation Treatment Guide 2011 of Stoke in China* (15) and *Practical Rehabilitation* (16). The members of the rehabilitation team examined the patient to determine the nature and extent of the disorder. The rehabilitation team also held review meetings to integrate patient care, formulate a rehabilitation plan, and implement treatment. According to the specific conditions of patients, they were trained to sit, balance, stand, shift their center of gravity, walk, feed themselves, change clothes, or go to the bathroom. They also received systemic coordination training, which included balancing, practical walking, using a walking stick, and going upstairs and downstairs.

#### Acupoints

The optimal acupoint scheme for this test was determined based on a consensus of acupuncture experts and *Acupuncture and Moxibustion Science* (17). Treatment for arm 1 was based on an acupoint summary from ancient literature (18, 19) and treatment for arm 2 followed the composition of the acupuncture points in the textbook. The most frequently used twelve acupoints in the ancient books and *Acupuncture and Moxibustion Science* were determined based on the consensus of acupuncture experts. In arm 1, these acupoints included GV20 (*baihui*), GV26 (*shuigou*), PC9 (*zhongchong*), ST6 (*jiache*), ST4 (*dicang*), LI15 (*jianyu*), LI11 (*quchi*), LI4 (*hegu*), GB30 (*huantiao*), GB31 (*fengshi*), GB34 (*yanglingquan*), and GB39 (*xuanzhong*). In arm 2, the acupoints used were GV20 (*baihui*), PC6 (*neiguan*), LI11 (*quchi*), LI10 (*shousanli*), SJ5 (*waiguan*), LI4 (*hegu*), GB30 (*huantiao*), ST36 (*zusanli*), GB34 (*yanglingquan*), SP6 (*sanyinjiao*), ST41 (*jiexi*), and LR3 (*taichong*). Bilateral acupoints were needled on PC9 and PC6, while all the remaining acupoints were selected from the affected side.

#### Acupuncture Intervention

The acupuncture treatment was performed by 16 different acupuncturists (with between 2 and 7 years of experience) at three different hospitals. Seven of the acupuncturists had Bachelor's level education, six had Master's degrees, and three had doctorates and all of them were registered Chinese medicine practitioners in China. All the researchers and acupuncturists were required to undergo a 4-day training session before the trial.

Appropriate positioning of the needles is crucial to obtaining good acupuncture results. Patients in this study were asked to lie in a lateral position with the affected side facing up. The hemiplegic shoulder stretched forward, with the shoulder joint flexed at 90° and the paraplegic upper limb resting on a pillow at a 100° angle to the trunk. The elbow was straightened, with arm, wrist, and fingers extended and palm facing up. Next, the paraplegic side of the lower extremity was placed on the pillow, revealing a step-like shape (with hips and knees flexed). The disposable sterile acupuncture needles used had varying specifications (0.30 mm × 25 mm, 0.30 mm × 40 mm, 0.30 mm × 50 mm, or 0.30 mm × 75 mm). Conventional disinfection with 75% alcohol was employed after acupuncture point positioning in accordance with *Standard Acupuncture and Moxibustion Positioning by the WHO* (20). During the acupuncture session, patients would feel soreness, numbness, distension or heaviness around the point, or an electric shock feeling during needling, indicating effective needling. To stimulate needle sensation, the needles were inserted flat and backward, 25 mm into DU20, and 8–15 mm obliquely and upward into DU26. Then, a reducing method was used for 30 s, rotating at a small amplitude and high frequency. The other acupoints utilized the reinforcing-reducing method 2.5 mm deep into PC9; 15–20 mm vertically into ST6, ST4, GB39, ST41, and LR3; 15–25 mm perpendicularly into LI4, PC6, and SJ5; 25–40 mm obliquely and downward into LI15; 25–40 mm perpendicularly into LI11, GB34, GB31, LI10, ST36, and SP6; and 50–70 mm perpendicularly into GB30. After insertion, the needles were left *in situ* for 30 min. The course of treatment was five times a week for consecutive 2 weeks. All the necessary precautions were taken to prevent any adverse events that occurred during treatment (e.g., fainting and broken needles). All the adverse events were recorded.

#### Control Intervention

The control group used the basic treatment plan, including baseline medications and rehabilitation training. The rehabilitation plan is mainly determined by the rehabilitation team.

### Outcomes

The treatment effect of this trial was evaluated by three scales at different time points (baseline = T0, week 1 = T1, week 2 = T2, and follow-up = T4). The primary outcome was expressed using the National Institutes of Health Stroke Scale (NIHSS). It ranged from 0 to 40 and included 11 items: consciousness, limb movement, eye movement, vision, facial paralysis, feeling, language, dysarthria, and neglect. It also measured the improvement in neurological function of patients (i.e., the lower the score, the lesser the patient's neurological deficit). The secondary outcomes were the Barthel Index (BI) scale and the Modified Ashworth Scale (MAS). The BI scale is typically used to assess patient self-care ability and if the score is below 20, this means that the patient's self-care ability had been seriously impaired. A score of over 60 indicated patients had been able to care for themselves. The improvement and difference in muscle tension within the three groups were determined by the changes in the Modified Ashworth Scale, both before and after treatment—the higher the score, the higher the patient's muscle tone. Since returning to the hospital after discharge is not an easy task for patients with stroke, only BI data for these three observations could be obtained in a remote setting such as a telephone conversation. Thus, only BI was measured four times.

### Sample Size

Referring to previous studies (21, 22), after 15 days of treatment, the NIHSS score variances before and after treatment for simple stroke rehabilitation was−3.95 ± 4.05, for acupuncture combined with rehabilitation was −6.07 ± 3.99, and for another acupuncture group combined with rehabilitation was −5.93 ± 4.08. Setting α to 0.05, β to 0.1, and substituting the sample size formulae of one-way ANOVA, the results showed that 76 patients were necessary for each group to complete the trial. Considering the similar curative effect between the two treatment groups, the two treatment groups need to be compared with the control group separately. According to Zhou (21), after 15 days of treatment, the NIHSS score for simple stroke rehabilitation was 8.86 ± 3.15 and the NIHSS score for acupuncture combined with stroke rehabilitation was 7.54 ± 2.68. Setting α to 0.05, β to 0.1, and substituting the sample size formulae of the two independent samples *t*-test, the results showed that 105 patients were necessary for each group to complete the trial. Assuming a 20% dropout rate per group, each group was adjusted to 126, with a total of about 378 patients. Finally, we determined that a sample size of at least 378 can better meet the statistical requirements.

### Statistical Analysis

An intention-to-treat (ITT) population was surveyed before the analyses, which included those patients undergoing baseline assessment and at least one evaluation after treatment. The NIHSS, the BI, and the MAS scores, along with changes over time, were compared by using repeated measures designed for the three groups. We also employed ANOVA to define the among-group differences at each measurement time point. The chi-square test or Fisher's exact test was used to analyze categorical variables, including categorical baseline variables and incidence of adverse events. The last observation carried forward (LOCF) was used in the ITT analysis to address missing data due to attrition and shedding. Statistical significance was defined as a two-tailed *P* < 0.05. Statistical analysis was performed with PASW version 20.0 (IBM SPSS Incorporation, Armonk, New York, USA).

## Results

### Baseline Data

**Figure 1:**
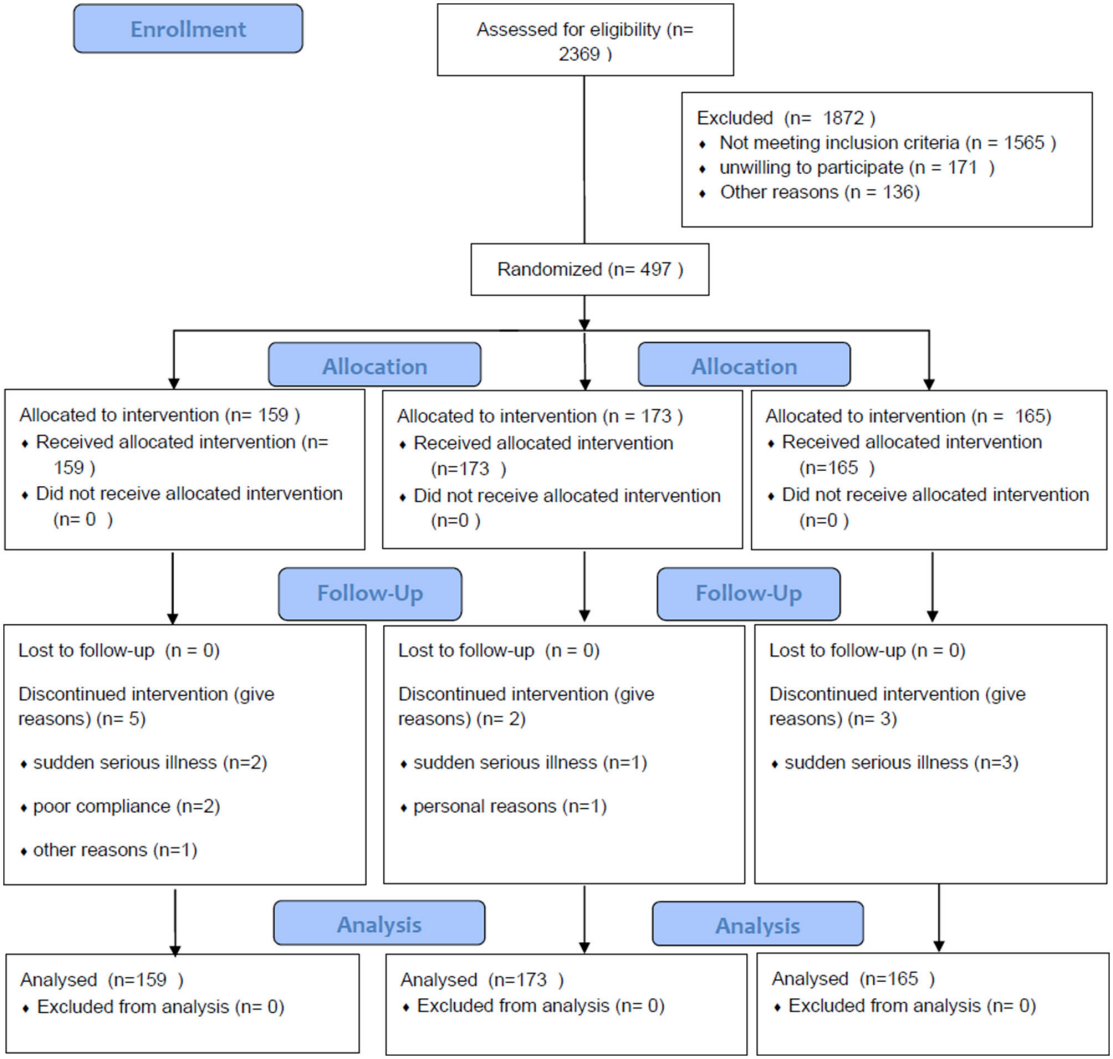
Flow diagram showing the study design.

Between July 2016 and July 2017, a total of 2,369 patients were assessed for eligibility. 497 of them participated in the trial (Guangzhou Hospital of Chinese Medicine = 200, First Affiliated Hospital of Guangzhou University of Chinese Medicine =247, and Liwan District Hospital of Chinese Medicine = 50). They were randomly divided into the three groups (arm 1 = 159, arm 2 = 173, and the control group = 165). Ten patients dropped out before the completion of the trial, six patients stopped treatment because of sudden serious diseases such as heart failure or lung infection, and three patients could not comply with the treatment schedule. One patient was lost due to a change in the contact phone number (Figure 1).

Table 1 shows the basic information for patients involved in this study, totaling 311 men and 186 women, all aged around 65 years. Most of the patients were not college-educated and most lived in urban areas. Twenty-five point eight percentage of patients had a history of smoking and 15.5% of patients had a history of moderate to heavy alcohol consumption. No statistical significance was found in the general variables (e.g., sex, age, education, and career) among the three groups. No statistically significant difference was found in ischemic stroke characteristics (disease course, number of strokes, infarct size, and family history, see Table 2).

**Table 1:** Baseline characteristics of patients (*n* = 497).

**General information**	**Arm 1 (*n* = 159)**	**Arm 2 (*n* = 173)**	**Control group (*n* = 165)**	**Total**
Sex				
Male	107(67.3)	107(61.8)	97(58.8)	311
Female	52 (32.7)	66(38.2)	68(41.2)	186
Age	64.3± 8.3	65.2 ± 8.5	65.4 ± 6.9	64.98 ± 0.3
Education				
Primary school or illiterate	31(19.5)	31(18.0)	36(22.0)	98(19.8)
Secondary school	53(33.3)	68(39.5)	62(37.8)	183(37.0)
High school	61(38.4)	61(35.5)	61(35.5)	176(35.6)
University	14(8.8)	12(7.0)	54(32.9)	38(7.7)
Master's or above	0(0)	0(0)	0(0)	0(0)
Career				
Office clerk	23(14.5)	23(13.3)	18(10.9)	64(12.9)
Manual worker	22(13.8)	27(15.6)	31(18.8)	80(16.1)
Student	0(0)	0(0)	0(0)	0(0)
Retired	84(52.8)	99(57.2)	87(52.7)	270(54.3)
Unemployed	11(6.9)	13(7.5)	12(7.3)	36(7.2)
Other	19(11.9)	11(6.4)	17(10.3)	47(9.5)
Residence				
Town	132(83.0)	150(86.7)	137(83.0)	419(84.3)
Village	27(17.0)	23(13.3)	28(17.0)	78(15.7)
Smoking				
None	114(71.7)	123(71.1)	132(80.0)	369(74.2)
Former smoker	31(19.5)	27(15.6)	20(12.1)	78(15.7)
Current smoker	14(8.8)	23(13.3)	13(7.9)	50(10.1)
Alcohol				
None	130(81.8)	151(87.3)	139(84.2)	420(84.5)
Former user	19(11.9)	13(7.5)	17(10.3)	49(9.9)
Current user	10(6.3)	9(5.2)	9(5.5)	28(5.6)

**Table 2:** Ischemic stroke characteristics.

**General information**	**Arm 1 (*n* = 159)**	**Arm 2 (*n* = 173)**	**Control group (*n* = 165)**	**F/ *X*^2^**	***P***
Disease course					
Month	3.49 ± 3.11	4.12 ± 4.10	3.83 ± 3.25	1.097	0.335
Times of stroke	1.26 ± 0.53	1.25 ± 0.44	1.28 ± 0.50	0.134	0.857
Infarct size					
Lacunar	28(17.7)	29(17.8)	45(27.4)	6.467	0.373
Multiple	81(51.3)	87(53.4)	78(47.6)		
Large area	16(10.1)	15(9.2)	13(7.9)		
Other	33(20.9)	32(19.6)	28(17.1)		
Family history of stroke					
No	129(81.1)	134(77.9)	123(74.5)	4.635	0.324
Unknown	26(16.4)	36(20.9)	35(21.2)		
Yes	4(2.5)	2(1.2)	7(4.2)		

Mean scores and changes in the NIHSS, the BI, and the MAS at different time points in the three study arms are shown in Table 3. In the statistical analysis of the above three items, no difference was found among the three groups in the repeat measurement design.

**Table 3:** Scores and changes for the National Institutes of Health Stroke Scale (NIHSS), the Barthel Index (BI), and the Modified Ashworth Scale (MAS) in the three study arms (x ± SD).

	**Group**	**T0=baseline**	**T1 = 1 week**	**T2 = 2 weeks**	**Follow up = 4 weeks**
NIHSS	Arm 1	7.13 ± 4.91	5.98 ± 3.72	4.59 ± 3.47^a^^b^	
	Arm 2	6.88 ± 4.08	6.11 ± 4.01	4.88 ± 4.11	
	Control group	6.98 ± 3.93	6.62 ± 4.03	5.81 ± 4.11	
BI	Arm 1	54.94 ± 27.01	60.00 ± 27.92	67.50 ± 28.22^a^	69.54 ± 27.69^a^
	Arm 2	57.82 ± 27.40	61.84 ± 27.58	69.26 ± 8.64^c^	71.15 ± 28.73^c^
	Control group	54.34 ± 27.52	56.62 ± 28.31	60.52 ± 28.69	62.68 ± 28.69
MAS	Arm 1	2.17 ± 1.32	2.03 ± 1.18	1.78 ± 0.99	
	Arm 2	2.34 ± 1.38	2.06 ± 1.24	1.80 ± 1.06	
	Control group	2.18 ± 1.37	2.09 ± 1.24	1.96 ± 1.17	

a *There were significant differences between the three groups*.

b *A comparison between arm 1 and the control group was statistically significant*.

c *A comparison between arm 2 and the control group was also statistically significant*.

**Figure 2:**
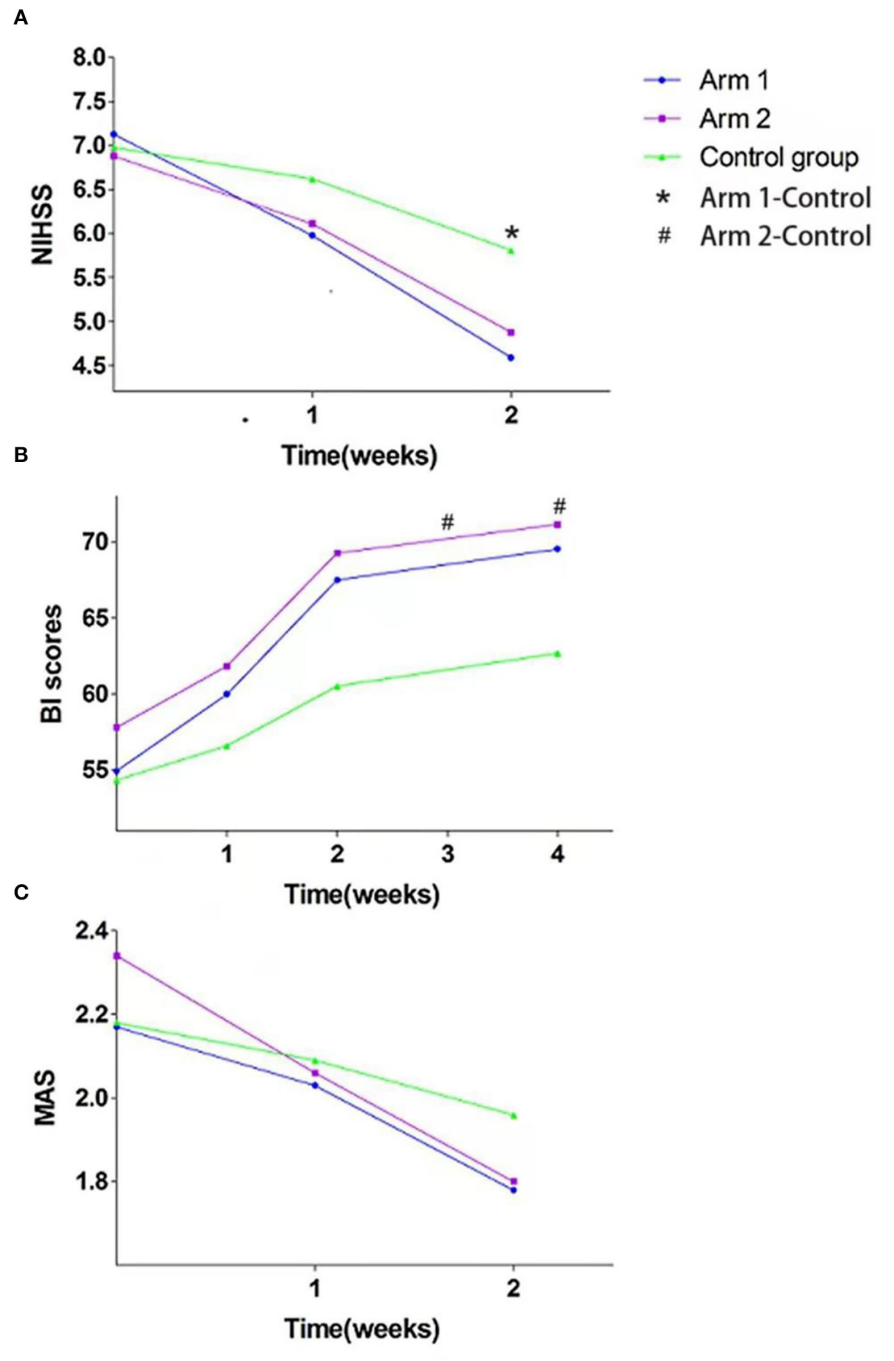
Scores of the **(A)** National Institutes of Health Stroke Scale (NIHSS), **(B)** the Barthel Index (BI), and **(C)** the Modified Ashworth Scale (MAS) in the three groups. *, # denotes significant difference.

The NIHSS scores of the three groups at each time point were analyzed and the results showed no significant difference in the NIHSS scores, at either T0 or T1 (*P* > 0.05). However, the difference between the NIHSS scores at T2 was statistically significant (*P* = 0.014). Further between-group comparisons found that there was a statistically significant difference between arm 1 and the control group (*P* = 0.017). As shown in Figure 2A, the NIHSS score in arm 1 had the largest reduction.

During the treatment and follow-up period, the BI scores of the three groups were accentuated. ANOVA showed that there were no significant differences either at T0 or T1 (*P* > 0.05), while the difference between the BI scores at T2 and follow-up was statistically significant (*P* = 0.014 and *P* = 0.017). Between-group comparisons showed a statistically significant difference in the arm 2 BI scores at T2 (*P* = 0.016) and follow-up (*P* = 0.020). However, we found no other marked differences at other time points (see Figure 2B).

Although there was an obvious decrease in the MAS scores during the treatment period, no statistically significant difference across the three groups was found at any time point (see Figure 2C).

## Safety Assessment

Over the course of acupuncture treatment for the three groups, there were no obvious changes in blood counts, routine urine tests, liver function, renal function, or heart enzymes. There were 5 adverse events in arm 1, 7 adverse events in arm 2, and no adverse events in the control group. Of these adverse events, nine were cases of bleeding, and three were cases of sticking of the needle. We stopped the bleeding by applying pressure with a sterile swab for 10 s and gently poking the needle handle, thus facilitating needle removal, tapping the needle handle to relieve muscle tension, and eliminating needle stagnation. No patients withdrew from the trial due to adverse events.

## Discussion

We have successfully completed a multicenter randomized controlled clinical trial on the effectiveness and safety of acupuncture for ischemic stroke rehabilitation. Acupuncture and moxibustion, as traditional treatment methods of traditional Chinese medicine, have long been confirmed for patients with stroke. It has the advantages of wide indications, obvious curative effect, convenient operation, economic safety, and so on. Acupuncture and moxibustion can improve the elasticity of patients' cerebral arteries, reduce their tension, dilate blood vessels, increase blood flow, promote the establishment of collateral circulation, improve the blood pressure circulation of the brain, and promote the repair of brain tissue. Studies by foreign scholars have also confirmed that acupuncture and moxibustion can maximize the activation of the motor cortex and promote the recovery of motor function by improving the plasticity of motor function. This method has been used in the treatment of stroke in China and East Asia for thousands of years. We found that compared with the control group, arm 1 and arm 2 were clinically effective and safe. The study has shown that acupuncture treatment after ischemic stroke can generate neuroprotective and neuroregenerative effects that increase cerebral blood flow, regulate oxidative stress, maintain blood–brain barrier integrity, inhibit apoptosis, and increase growth factor production (23). This may be the basis for acupuncture treatment of ischemic stroke. At present, ischemic stroke treatment is mostly performed in the setting of stroke units, which are considered to be the most effective way to deal with the disease (24–26). In many stroke units, various therapies and techniques are combined to provide patients with therapeutic medications and physical, language, and psychological rehabilitation, and also health education (27). In China, acupuncture also plays an indispensable role in the clinical rehabilitation of stroke (28, 29).

In stroke diseases, the occurrence and gradual development of limb paralysis in patients will lead to damage to the cortex and problems in the basal ganglia and brain cadres. Hemiplegia is mainly due to the damage to patients' upper motor neurons, which leads to related diseases. When the upper motor neuron is damaged, the inhibition of pivot reflex will be relieved and large muscle tension will be generated; in the case of combined with other diseases, it will hinder the movement of patients. From the perspective of traditional Chinese medicine, the limb hemiplegia of patients with stroke is the category of “spasm” or “contracture,” which needs to dredge the blood stasis in time, i.e., acupuncture points and act on the patient's central nervous system through acupuncture and moxibustion, so as to weaken the promoting effect of the descending central nervous system of the patient's spinal cord and to alleviate the symptoms of muscle spasm.

GV20 is located on the top of the head and can adjust the medullary sea. Modern studies have confirmed that stimulating Baihui acupoint can inhibit the oxidative stress state of the chronic stress rat model, improve cerebral hypoxia and blood circulation, enhance brain antioxidant capacity, delay neuronal apoptosis, and have a brain-protective effect on depressed rats. DU26 belongs to the category of thirteen ghost acupoints, also known as the Shuigou acupoint. It is the intersection of the Yangming Meridian of hand and foot and the governor vessel. Modern studies have proved that the Shuigou point contains branches of the facial nerve and trigeminal nerve, with a rich blood supply and a rich distribution of nerve fibers. Stimulating this point can improve cerebral blood supply. According to the study results, the function of the above acupoints and the correctness of acupoint selection were confirmed.

The NIHSS is an important scale for stroke assessment. It comprehensively evaluates the consciousness, movement, sensation, and advanced neurological function of patients with stroke. In this trial, arm 1 was superior to the control group in treating neurological deficits; however, there was no marked difference between arm 2 and the control group. The reduction in the NIHSS score predicts that the degree of neurological impairment was better than before, indicating that the treatment was beneficial to the disease recovery. Previous studies have shown that GV20-based acupoint combination together with rehabilitation is more effective than simple rehabilitation in reducing the NIHSS scores after eight courses of treatment (21), largely owing to the fact that these acupoints can balance the body's *yin* and *yang* and dredge the *qi* and blood of the meridians. In terms of improving patient self-care ability, the effect of arm 2 was better than that of the control group, but there was no significant difference between arm 1 and the control group. Increased BI scores mean that patients have improved their lives in terms of eating, dressing, walking, and going to the bathroom. While a multicenter RCT revealed that the BI scores of patients with subacute stroke were significantly higher after 6 months of body and scalp acupuncture treatment, no obvious improvements were found when compared with the rehabilitation group (30). In the authors' opinion, this negative result was due to the acupoints in the program deviating from the viewpoint of TCM syndrome differentiation. Thus, we should not only focus on the proximity to afferent nerve fibers but also select points according to the specific conditions of patients. The acupoints in this study were derived from a combination of multiple clinical trials and the experience of several seasoned acupuncturists. The two treatment groups exerted a similar effect to that of the control group in terms of improving muscle tension. However, many experiments have shown that acupuncture or electroacupuncture combined with rehabilitation can improve muscle tone and release spastic limbs (31–34). In our opinion, this is likely related to insufficient treatment or observation time. Further clinical studies are needed to confirm this hypothesis.

In designing the trial's scheme, considering the adjustment of multicentric effect and multiple independent variable effects in statistical models, we determined to include more samples besides 378 to obtain more statistical power. During the trial process, the control group also received basic treatment and rehabilitation without violating ethics.

This experiment is different from other similar trials. One is a multicenter participation and a large sample size. The other is a selection of acupoints by not only referring to ancient books but also by paying attention to modern literature and comprehensively evaluating the curative effect of acupuncture on stroke. This experiment proves that acupuncture has positive significance for the recovery of patients with ischemic stroke and promotes the popularization and application of acupuncture in patients with stroke.

## Limitations of This Study

Although this trial is one of the few random, multicenter, and large-sample trials of acupuncture treatment for stroke (35), some limitations were observed and require attention in the future. First, the course of treatment was relatively short. After two courses of treatment, acupuncture has achieved a good curative effect and positive effects may emerge in the treatment group with the passage of treatment time. Second, our study was not double-blinded. Although clinical trials on acupuncture are mostly conducted in a single-blind state, the possible risk is that the acupuncturists are affected by them after communicating with patients, that is, the experimenter's own preferences are passed onto the participants and cause experimental deviation. This may have led to treatment outcome bias.

## Conclusion

Acupuncture treatment for ischemic stroke demonstrated better recovery results than the simple rehabilitation group. Specifically, group 1 was better than the control group in improving the degree of neurological impairment, while group 2 was preferred in improving patients' ability of daily living. But there was no difference between the effects of the two acupuncture groups.

## Data Availability Statement

The raw data supporting the conclusions of this article will be made available by the authors, without undue reservation.

## Ethics Statement

The studies involving human participants were reviewed and approved by the Ethics Committee of the Guangzhou Hospital of Chinese Medicine (Reference No. 2016NK001). The Affiliated TCM Hospital of Guangzhou Medical University. The patients/participants provided their written informed consent to participate in this study.

## Author Contributions

LLi, CC, and GL designed and led the study. WZ, DT, XW, WL, and SL analyzed and interpreted the patient data to generate the study results. LLu and LX drafted the manuscript. All authors have read and approved the final version of the manuscript.

## Conflict of Interest

The authors declare that the research was conducted in the absence of any commercial or financial relationships that could be construed as a potential conflict of interest. The reviewer XT declared a shared parent affiliation with the authors GL and SL to the handling editor at the time of review.

## Publisher's Note

All claims expressed in this article are solely those of the authors and do not necessarily represent those of their affiliated organizations, or those of the publisher, the editors and the reviewers. Any product that may be evaluated in this article, or claim that may be made by its manufacturer, is not guaranteed or endorsed by the publisher.

## Abbreviations

NIHSS: National institutes of Health Stroke Scale
BI: Barthel index
MAS: Modified Ashworth Scale
STRICTA: Reporting Interventions in Clinical Trials of Acupuncture
WHO: World Health Organization.
